# Gold(I)-catalyzed enantioselective cycloaddition reactions

**DOI:** 10.3762/bjoc.9.264

**Published:** 2013-10-30

**Authors:** Fernando López, José L Mascareñas

**Affiliations:** 1 Instituto de Química Orgánica General, CSIC and Centro Singular de Investigación en Química Biolóxica e Materiais Moleculares (CIQUS). Universidade de Santiago de Compostela, 15782, Santiago de Compostela, Spain; 2 Departamento de Química Orgánica, Centro Singular de Investigación en Química Biolóxica e Materiais Moleculares (CIQUS). Universidade de Santiago de Compostela, 15782, Santiago de Compostela, Spain

**Keywords:** alkyne, allene, asymmetric catalysis, cycloaddition, enantioselective, gold, gold catalysis

## Abstract

In recent years there have been extraordinary developments of gold(I)-catalyzed enantioselective processes. This includes progress in the area of cycloaddition reactions, which are of particular interest due to their potential for the rapid construction of optically active cyclic products. In this article we will summarize some of the most remarkable examples, emphasizing reaction mechanisms and key intermediates involved in the processes.

## Introduction

In the past decade, there have been extraordinary advances in the development of novel stereoselective gold(I)-catalyzed transformations [1–10]. In this context, enantioselective gold catalysis has been identified as a particularly challenging goal because the linear two-coordination mode of gold(I) complexes and the out-sphere π-activation usually associated to carbophilic gold catalysts [11] places ligands far from the reacting centers, thus limiting the capacity to transfer asymmetry [12].

A number of strategies to tackle this problem have been developed, most of them based on the use of a new type of chiral gold complexes. This resulted in a number of gold-catalyzed enantioselective transformations in the past years, including hydrogenations, aldol reactions, 1,3-dipolar cycloadditions, and cyclizations [13–15]. Other gold-promoted asymmetric induction strategies rely on the use of chiral counterions. Indeed, it has been shown that a tight chiral ion pair with the gold cation is able to induce excellent levels of asymmetry in certain cyclizations [16].

Cycloaddition reactions are very important synthetic processes that allow the transformation of simple acyclic precursors into complex cyclic or polycyclic adducts in a rapid and efficient way [17–18], usually providing a rapid increase in skeletal and stereochemical complexity. Moreover, cycloadditions are atom economical, and usually take place with high levels of regio- and stereocontrol. Especially relevant in terms of synthetic practicality are cycloadditions which are catalyzed by transition metal complexes [19–23]. In particular, gold(I) complexes, owing to their high carbophilicity, low oxophilicity and high oxidation potential between gold(I) and gold(III) have shown a unique potential to unveil novel types of chemoselective and stereoselective cycloadditions involving alkynes, allenes or alkenes [24–26].

A lot of interest has been directed to the development of these cycloaddition processes in an enantioselective manner, so that the resulting cyclic products could be obtained in an optically pure fashion [27]. Herein, we describe the most relevant types of enantioselective cycloaddition reactions based on the use of carbophilic gold(I) complexes. We do not consider cycloaddition reactions in which the gold complex acts more like a conventional Lewis acid rather than by activating π-bonds [28–32]. The reactions included in this review are classified according to the type of key reactive gold intermediates that formally participate in the cycloaddition. Thus, we will present cycloaddition processes (i) with a gold–carbene intermediate, (ii) involving an allene activation to generate a gold–allyl cationic intermediate and, (iii) in which the proposed key reactant is a vinylgold zwitterionic species.

## Review

### Cycloadditions involving gold–carbene intermediates

**Figure 1:**
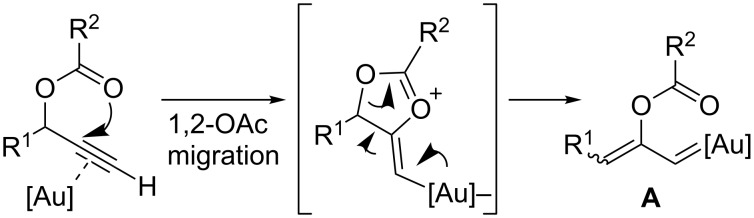
Gold-promoted 1,2-acyloxy migration on propargylic systems.

Gold–carbene species are frequent intermediates in gold-catalyzed reactions, in particular those involving alkynes [33–37]. Gold(I) catalysts bind chemoselectively to C–C triple bonds, promoting the attack of different types of nucleophiles on these electrophilic species. Depending on the particular system, the resulting vinylgold intermediates can be externally trapped or evolve to reactive carbene species. This is the case for propargyl esters (Figure 1), as these systems usually undergo 1,2 or 1,3-acyloxy migrations in the presence of gold catalysts. Such migrations proceed via a nucleophilic intramolecular attack of the carboxy moiety on the activated alkyne. 1,2-Migration of the ester affording a gold–carbene of type **A** is usually preferred when a terminal alkyne is used [38–40].

Based on this concept, several groups have shown that the resulting carbenoid intermediates of type **A** can engage in different types of cycloaddition reactions with diverse C–C unsaturated systems. Here, we discuss the systems for which an enantioselective variant has been developed.

**Scheme 1:**
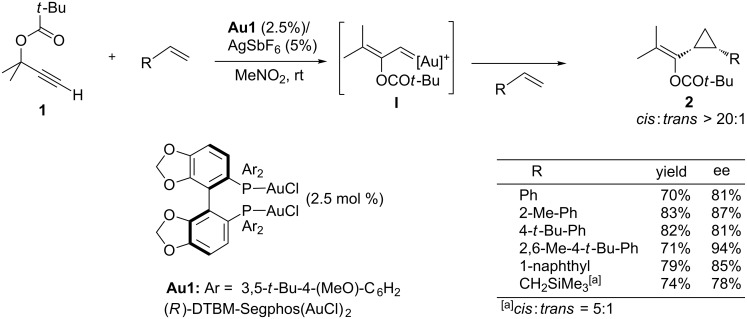
Gold-catalyzed enantioselective intermolecular cyclopropanation.

In 2005, Toste and coworkers described one of the first gold-catalyzed enantioselective processes that could be formally categorized as a [2 + 1] cycloaddition. In particular, they showed that it is possible to trap the intermediate gold–carbenes resulting from a 1,2-acyloxy migration in propargyl esters such as **1**, with external alkenes (e.g. vinylarenes), to give cyclopropane products [41]. The racemic variant of the method, which employs Ph_3_PAuCl/AgSbF_6_ as a catalyst, predominantly affords *cis*-cyclopropane adducts of type **2**, and tolerates a wide range of olefin substituents. Importantly, the authors demonstrated that the process could also be rendered enantioselective by using a chiral bisgold complex derived from DTBM-Segphos (**Au1**). High or even very high levels of enantioselectivity could be achieved when the propargylic system features sterically demanding esters such as pivaloates, or the alkene component presents large aromatic substituents. In all these cases the reaction afforded the *cis* isomer with high diastereoselectivity. Moreover, the enantioselective cyclopropanation was not limited to arylated olefins (e.g. styrenes), but allyltrimethylsilane also participated in the process, producing the corresponding silylmethyl cyclopropane as a 5:1 mixture of *cis*:*trans* isomers with a good 78% ee (Scheme 1).

**Scheme 2:**
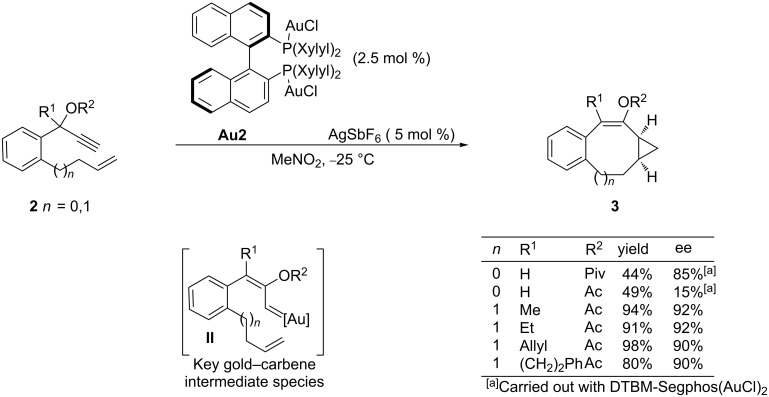
Gold-catalyzed enantioselective intramolecular cyclopropanation.

In 2009, the same group extended the utility of this asymmetric cyclopropanation reaction to an intramolecular process that allows the enantioselective synthesis of polycarbocyclic products embedding seven or eight-membered rings [42]. Curiously, the catalytic system based on DTBM-Segphos, which was particularly successful in the abovementioned intermolecular cases, only provided good enantioselectivities in the case of systems affording products with seven-membered rings (**3**, *n* = 0, Scheme 2). For the eight-membered counterparts, the authors found that a related bisphosphine–gold catalyst, Xylyl-Binap(AuCl)_2_/AgSbF_6_ was more efficient, facilitating good yields of the corresponding products and enantioselectivities between 75 and 92%. In these cases, the bulky pivaloate at the propargylic position led to lower enantioselectivites than a less congested acetate group, showcasing that a fine-tuning of the catalyst and substrate is required to achieve excellent enantioselectivities.

**Scheme 3:**
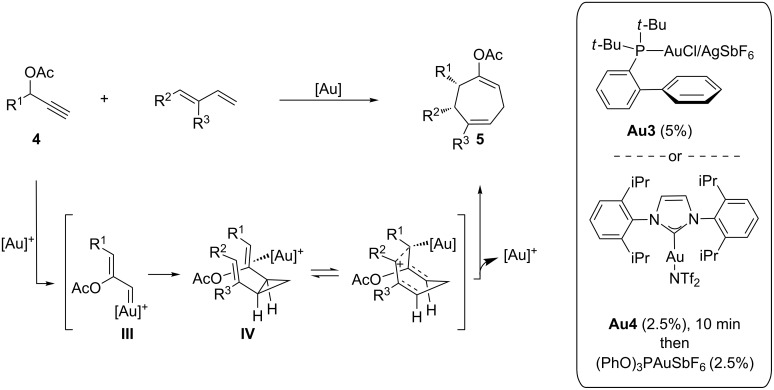
Gold-catalyzed cyclohepta-annulation cascade.

More recently, Nevado and co-workers have shown that propargyl acetates **4** react with 1,3-dienes in the presence of a gold catalyst to give good yields of cycloheptadiene products of type **5**; thus the process could be formally considered as a [4 + 3] annulation. A possible mechanism would involve a gold-mediated 1,2-acyloxy migration of the propargyl ester to generate a gold–carbene species **III** which cyclopropanates a C–C double bond of the diene to form a *cis*-cyclopropane intermediate **IV**. A subsequent gold-catalyzed Cope rearrangement through a boat-like transition state would deliver the *cis*-2,3-disubstituted cycloheptenyl acetates of type **5** (Scheme 3) [43].

**Scheme 4:**
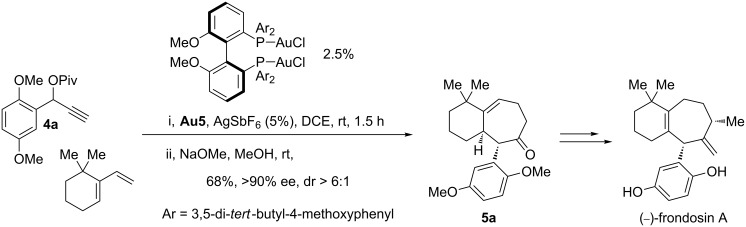
Application to the formal synthesis of frondosin A.

Although the process was essentially developed in a racemic fashion, by using **Au3**/AgSbF_6_ or a combination of **Au4** and (PhO)_3_PAuSbF_6_ (Scheme 3), the authors also demonstrated the feasibility of an enantioselective variant. Thus, treatment of pivaloate **4a** with 6,6-dimethyl-1-vinylcyclohexene in the presence of the chiral gold catalyst (*S*)-MeO-DTBM-Biphep(AuCl)_2_/AgSbF_6_, followed by in situ hydrolysis, allowed the construction of the basic bicarbocyclic core of frondosins (for example, **5a**), in 68% yield and 90% ee (Scheme 4). Since this bicyclic enone was previously elaborated into frondosin A and B, the approach represented a streamlined formal enantioselective synthesis of both molecules.

**Scheme 5:**
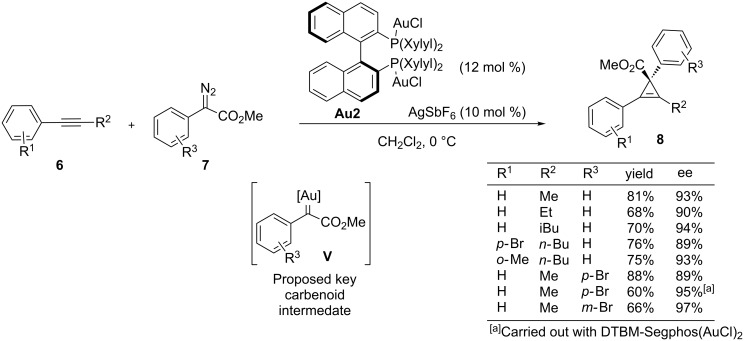
Gold(I)-catalyzed enantioselective cyclopropenation of alkynes.

Analogous to other transition metals from groups eight to eleven, gold has also demonstrated to be an efficient promoter of intermolecular carbene transfer reactions from diazo compounds to unsaturated systems such as alkenes or alkynes, resulting in cyclopropanation processes [44–45]. The development of an enantioselective variant of this type of reactions remained elusive until very recently, when Davies and co-workers reported a highly enantioselective cyclopropenation of internal alkynes **6** with aryldiazoacetates **7** [46]. In some cases the complex DTBM-Segphos(AuCl)_2_ (12%)/AgSbF_6_ (10%) provided the best performance. For most of the substrates, however, the chiral digold cationic complex XylylBinap(AuCl)_2_/AgSbF_6_ provided higher enantiomeric excesses and better yields of the desired cyclopropenes **8** (Scheme 5). The scope of the method encompasses a variety of aryl disubstituted alkynes **6** and several donor/acceptor aryldiazoacetates **7**.

**Scheme 6:**
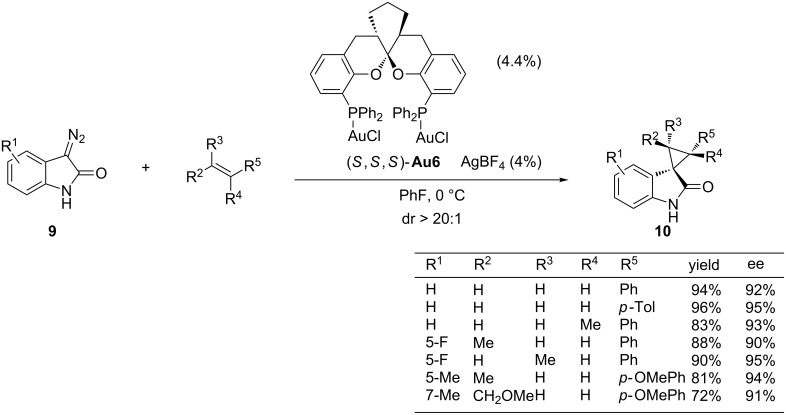
Enantioselective cyclopropanation of diazooxindoles.

In 2013, Zhou and co-workers reported another example of a highly enantioselective gold-catalyzed cyclopropanation reaction by using diazo compounds as a source of gold carbenes. In particular, the authors demonstrated that the chiral bisgold complex **Au6**, derived from the spiroketal bisphosphine ligand, was a very efficient promoter of the cyclopropanation between donor–acceptor diazooxindoles such as **9** and a broad range of alkenes (Scheme 6) [47]. The resulting spirocyclopropyloxindoles **10**, which are obtained in excellent yields and enantioselectivities, are appealing structures from a medicinal point of view. The scope of the alkene is quite remarkable, since not only monosubstituted and 1,1-disubstituted olefins participated in the cyclopropanation, but also 1,2-disubstituted alkenes (*cis* or *trans*), which previously failed with other chiral transition metal catalysts, providing excellent yields and very good levels of enantioselectivity.

### Cycloadditions initiated by activation of allenes

**Figure 2:**
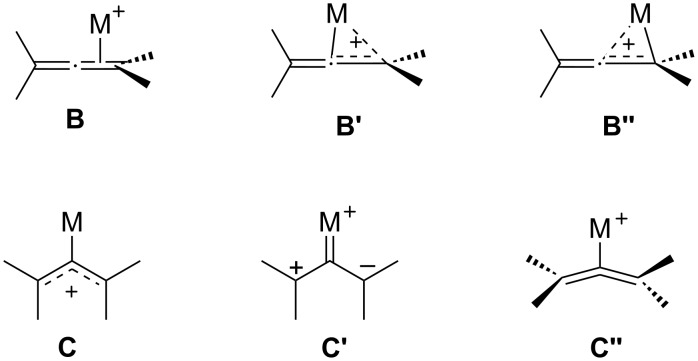
Proposed structures for gold-activated allene complexes.

Gold(I) catalysts can efficiently activate allenes in a highly chemoselective way, triggering the formation of allenyl cationic species. Different structures have been proposed to represent these gold-activated allene complexes, including η^2^-complexes (**B**, **B’** and **B’’**), planar σ-allylic cations **C**, zwitterionic carbenes **C’** or η^1^-bent allenes **C’’** (Figure 2) [48]. In some cases, experimental and theoretical evidence supports the participation of one of those structures, but in many other cases the precise nature of these species remains unknown. The gold-activated allene complexes have been shown to participate in a great variety of intra- and intermolecular cycloaddition reactions, including [4 + 3], [4 + 2], [3 + 3], [3 + 2] or [2 + 2] annulation processes [1–10,24–26]. Particular attention has been paid during the last years to the development of enantioselective versions of these processes by using chiral gold catalysts. In this section, we highlight the most important developments in this area.

**Scheme 7:**
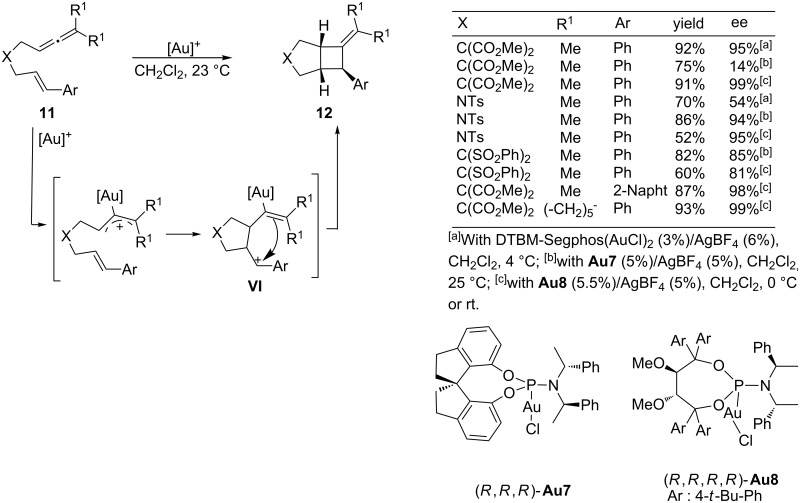
Gold-catalyzed enantioselective [2 + 2] cycloadditions of allenenes.

In 2007, Toste and coworkers described an intramolecular [2 + 2] cycloaddition of allenenes by using gold catalysts [49]. The proposed mechanism is based on the generation of a gold(I)-activated allene, which undergoes a cyclization to give a new carbocationic species of type **VI** (Scheme 7) [50–51]. A subsequent ring closure provides the observed bicyclo[3.2.0] systems of type **12**, featuring a four-membered carbocycle. These reactions, which are efficiently promoted by Ph_3_PAu^+^BF_4_ in their racemic variants, could be performed in a highly enantioselective manner with gold catalysts containing a DTBM-Segphos ligand. Enantioselectivities ranged from 54 to 96%, and seem to be highly dependent on the tether that links the allene and the alkene moieties. Thus, the reaction proved to be efficient for substrates containing tethers with geminal diesters, whereas those containing *N*-tosyl-based tethers led to significantly lower enantioselectivities. However, the same group more recently demonstrated that a chiral phosphoramidite–gold complex, such as **Au7**, could eventually solve this limitation, leading to a complementary reactivity to that exhibited by DTBM-Segphos(AuCl)_2_/AgBF_4_ in terms of substrate scope, and affording enantioselectivities up to 97% (Scheme 6) [51]. Additionally, Fürstner and co-workers have also demonstrated that a phosphoramidite–gold complex like **Au8**, containing an acyclic Taddol-based backbone, was also an excellent catalyst to perform the same type of [2 + 2] cycloadditions of allenenes **11** [52–53]. This catalyst performs equally well with carbon-based and nitrogen-based tethers, and excellent levels of asymmetric induction were obtained from a set of representative examples, including those with structural modifications at the allene and alkene sites (Scheme 7).

**Scheme 8:**
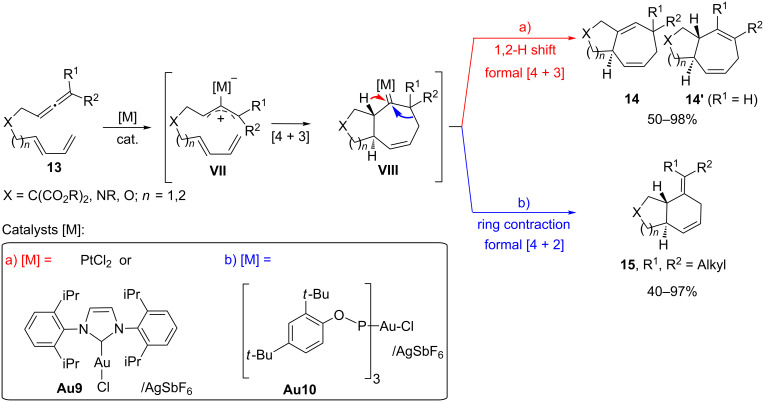
Gold-catalyzed allenediene [4 + 3] and [4 + 2] cycloadditions.

Early in 2008, our group demonstrated the possibility of using gold-activated allenes as allyl cation surrogates capable of participating in concerted [4C(4π) + 3C(2π)] cycloadditions with conjugated dienes [54], a process related to the classical oxyallyl cation [4 + 3] cycloadditions [55–57]. An initial screening demonstrated that PtCl_2_ was a good catalyst for promoting these intramolecular [4C + 3C] cycloadditions [54]. Further studies revealed that the scope of the methodology could be significantly broadened by using a gold catalyst such as **Au9**/AgSbF_6_ [58]. In general, the reactions are completely diastereoselective, affording bicyclo[5.3.0]decane products (**14** and/or **14’**) that result from an *exo*-like approach of the allyl cation to the diene. Curiously, by using a gold(I) catalyst bearing a π-acceptor phosphite ligand, such as **Au10**/AgSbF_6_, allenedienes disubstituted at the distal position of the allene lead to products formally arising from a [4 + 2] cycloaddition process (Scheme 8, b) [59–60]. Several experimental data as well as theoretical calculations suggested that both cycloadducts, **14** and **15**, arise from the same intermediate, the cycloheptanyl metal–carbene species **VIII**, which might evolve through a 1,2-hydrogen shift to give the seven-membered carbocycles **14** (Scheme 8, a), or by a ring-contraction process to give the cyclohexenyl products (Scheme 8, b) [60–64]. Therefore, the ligand at gold, as well as the type of substituents at the allene terminus, determine the fate of this carbene and hence the formation of the [4 + 3] (**14**) or [4 + 2] (**15**) cycloadducts.

**Scheme 9:**
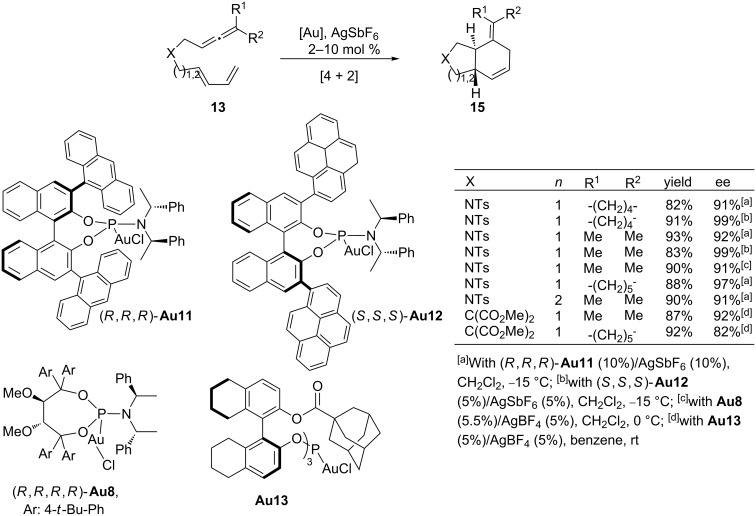
Gold-catalyzed enantioselective [4 + 2] cycloadditions of allenedienes.

The development of enantioselective versions of these two cycloadditions was actively pursued by several groups. The electronic similarity between phosphites and phosphoramidites suggested that chiral gold complexes of these highly modular monodentate ligands [65] could be good candidates to promote enantioselective variants of the [4 + 2] cycloaddition (Scheme 9, R^1^, R^2^ = alkyl groups). This turned out to be the case and, gratifyingly, we showed that it is possible to perform highly enantioselective allenediene [4 + 2] cycloadditions by using gold complexes derived from a bulky phosphoramidite equipped with anthracenyl substituents at the Binol ortho-positions (**Au11**, Scheme 9) [60]. Independently, Toste and co-workers reported that the related phosphoramidite–gold complex **Au12**, and the chiral gold catalyst **Au13**, derived from a *C*_3_-symmetric phosphite [66], were also able to induce excellent enantioselectivities in these [4 + 2] cycloadditions [67]. Moreover, Fürstner and co-workers also showed that the Taddol-based phosphoramidite–gold complex **Au8,** which was effective in the [2 + 2] cycloadditions of allenenes, was also able to induce good enantioselectivities in these higher order annulations (Scheme 9) [52].

**Scheme 10:**
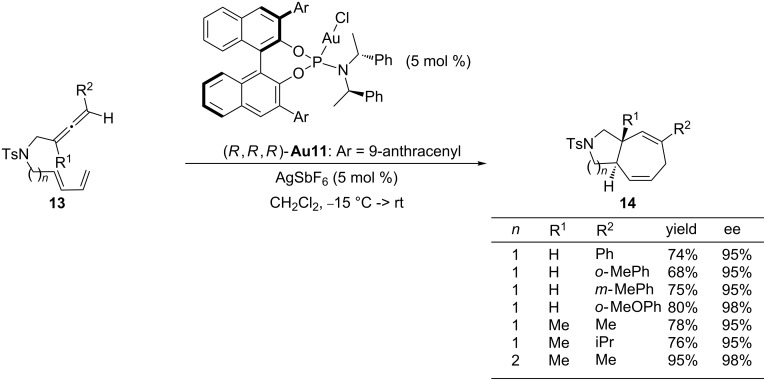
Gold-catalyzed enantioselective [4 + 3] cycloadditions of allenedienes.

Although π-acceptor ligands, such as phosphites or phosphoramidites favor the formation of the [4 + 2] adduct of type **15**, DFT calculations indicated that the activation barriers for the ring-contraction process leading to these six-membered systems, and the 1,2-H shifts that retain that seven-membered ring, are quite similar [60]. Therefore, it seemed plausible that this type of chiral catalysts could also provide an enantioselective entry to the seven-membered adducts of type **14**, provided that the ring-contraction route could be slightly deactivated. The viability of this hypothesis was validated by Mascareñas, López and co-workers, demonstrating that terminally monosubstituted allenes, which provide carbene intermediates that are less prone to undergo a ring contraction (**VIII**, Scheme 8), react with phosphoramidite–gold catalyst **Au11**/AgSbF_6_ to afford optically active, synthetically relevant bicyclo[5.3.0]decadiene and bicyclo[5.4.0]undecadiene skeletons **14** with good yields, complete diastereocontrol and excellent enantioselectivities (Scheme 10) [68]. The scope of this method, which constituted the first highly enantioselective intramolecular [4C + 3C] cycloaddition promoted by a transition metal complex, encompasses internally monosubstituted allenes, as well as disubstituted counterparts, offering a direct entry to 5,7 bicyclic systems including those with all-carbon quaternary stereocenters at the ring fusion.

**Scheme 11:**
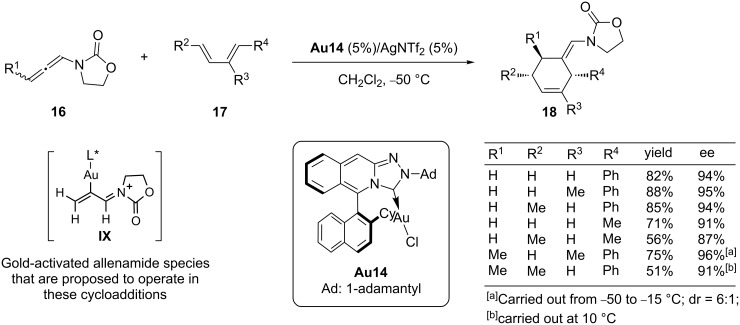
Gold-catalyzed enantioselective [4 + 2] cycloadditions of allenamides.

In contrast to the intramolecular counterpart, gold-catalyzed intermolecular cycloadditions of allenes have been scarcely studied. However, significant progress has been made in the last 4 years. While the development of an intermolecular [4 + 3] cycloaddition between allenes and dienes remains elusive, neither in a racemic nor enantioselective fashion [69], it has been shown that allenamides [70] or allenyl ethers [71] participate as two-carbon atom components in several gold-catalyzed [4 + 2] cycloadditions with dienes. The racemic version of the reaction between allenamides and dienes, which is efficiently promoted by AuCl or the cationic gold catalyst **Au9**/AgSbF_6_, was translated into a enantioselective version by using a novel chiral gold complex **Au14**, featuring a triazole unit embedded in a rigid axially chiral cyclic frame (Scheme 11) [72]. The catalyst generated from **Au14** and AgNTf_2_ was able to promote the [4 + 2] cycloaddition between allenamides **16** and conjugated dienes **17** with total regio- and stereoselectivity and excellent enantioselectivity. The method provides a versatile and practical approach to a variety of optically active cyclohexene products like **18**. Importantly, the scope of the asymmetric process is even wider than that of the racemic reaction [70], allowing the construction of six-membered adducts with up to three new stereogenic centers with complete diastereoselectivity and enantioselectivities of up to 91% ee (R^1^, R^2^ = Me, R^4^ = Ph, Scheme 11). With regard to the mechanism, it has been proposed that the activation of the allenamide by the gold catalyst provides a gold–allyl cationic species of type **IX**, which is the species undergoing the cycloaddition process with the diene [73].

**Scheme 12:**
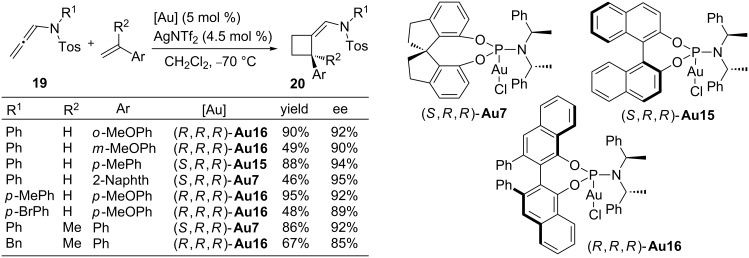
Enantioselective [2 + 2] cycloadditions of allenamides.

Simple alkenes do also react with gold-activated allenamides to provide cyclobutane products, formally resulting from a [2 + 2] cycloaddition. Thus, independent investigations by the groups of Mascareñas, González and Chen revealed that the gold-catalyzed cycloaddition between an allenamide and an appropriate alkene (e.g., enamide, enol ether or vinylarene) provides a variety of cyclobutanic systems in excellent yields. The optimum catalysts for the racemic processes include a phosphite–gold complex **Au10** and a biaryldi-*tert*-butylphosphine–gold complex (**Au3**) [74–76]. More recently, González and co-workers accomplished the enantioselective version of these annulations between sulfonyl allenamides and vinylarenes, providing a straightforward entry into optically active cyclobutanes [77]. Several chiral phosphoramidite–gold complexes, such as (*S*,*R*,*R*)-**Au7**, (*S*,*R*,*R*)-**Au15** and (*R*,*R*,*R*)-**Au16**, derived from Siphos, Binol and Vanol, respectively, provided excellent enantioselectivities, displaying useful complementarity in some of the cases. Importantly, the method also allowed the preparation of cyclobutanes **20** containing challenging quaternary carbon centers (Scheme 12).

**Scheme 13:**
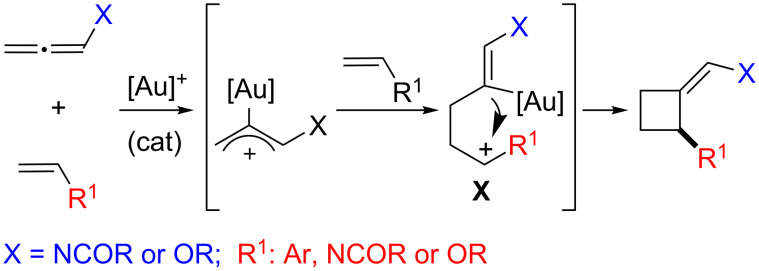
Mechanistic rational for the gold-catalyzed [2 + 2] cycloadditions.

From a mechanistic point of view, these [2 + 2] cycloadditions, either in racemic or asymmetric versions, have been proposed to proceed through a stepwise cationic pathway consisting of an initial activation of the allene to provide an Au–allyl cationic species, followed by a regioselective interception by the alkene to give a second cationic intermediate (**X**, Scheme 13) [73]. The substituent at the alkene (R^1^, Scheme 13), either an aryl, nitrogen or oxygen-group, plays a key role in the stabilization of this intermediate and therefore facilitates the overall process. Finally, a ring-closing process through attack of the vinylgold species to the stabilized carbocation yields the cyclobutane system and regenerates the catalyst.

**Scheme 14:**
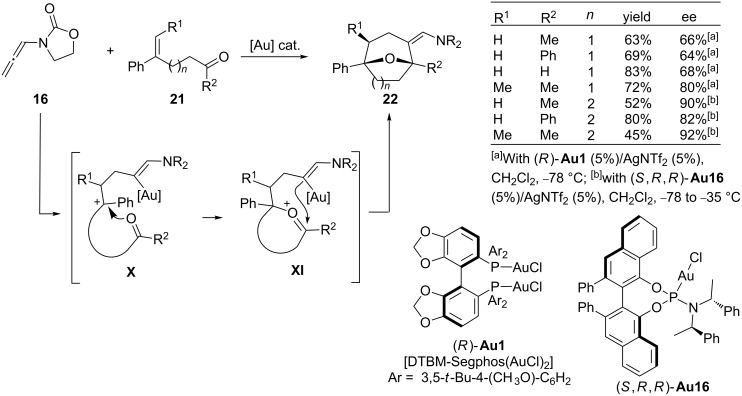
Enantioselective cascade cycloadditions between allenamides and oxoalkenes.

In view of this mechanistic proposal, Mascareñas and López recently developed a gold-catalyzed cascade cycloaddition between allenamides and carbonyl-tethered alkenes **21**, including an enantioselective variant, that provides synthetically appealing oxa-bridged seven, eight and even nine-membered rings (**22**, Scheme 14) [78]. The cascade process relies on the interception of intermediates of type **X** by an intramolecular carbonyl group, followed by the ring closing of the resulting oxonium intermediate **XI**. The reaction occurs at low temperatures by using only 1 mol % of a phosphite-based gold catalyst. The enantioselective version, which is catalyzed by the DTBM-Segphos complex **Au1**/AgNTf_2_, or the phosphoramidite–gold complex (*S*,*R*,*R*)-**Au16**/AgNTf_2_, provides the corresponding cycloadducts **22** with good or high enantioselectivites (Scheme 14). The method constituted the first direct catalytic and enantioselective entry to oxygen-bridged eight-membered carbocycles and one of the few methods that affords optically active cyclooctanes by means of a catalytic enantioselective process.

**Scheme 15:**
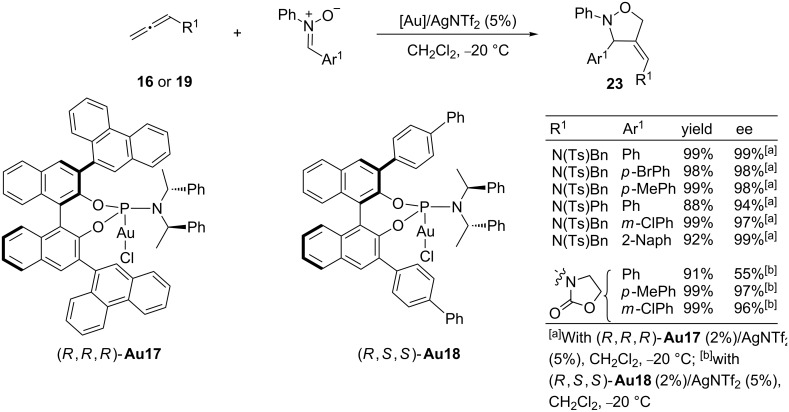
Enantioselective [3 + 2] cycloadditions of nitrones and allenamides.

Also recently, Chen and co-workers demonstrated that gold-activated allenamide species can participate in [3 + 2] cycloadditions with azomethine imines or nitrones under catalysis with Ph_3_PAuCl/AgOTf or Ph_3_PAuCl/AgNTf_2_ [79–80]. Moreover, for the latter cycloaddition with nitrones, the authors reported a enantioselective variant by using chiral phosphoramidite–gold complexes. In these cases, the binol-derived complexes, with aryl substituents at the 3 and 3’ positions, turned out to be the optimal systems, providing the corresponding 4-alkylidenylisoxazolidine derivatives **23** in high yields and excellent enantioselectivities. The highest enantioselectivities were obtained with (*R*,*R*,*R*)-**Au17** and (*R*,*S*,*S*)-**Au18**, which include 9-phenantrenyl or 4-biphenyl units in these binol *ortho*-positions, respectively (Scheme 15). Further derivatization of the products led to enantio-enriched 1,3-aminoalcohols.

### Other reactions involving zwitterionic alkenylgold intermediates

**Scheme 16:**
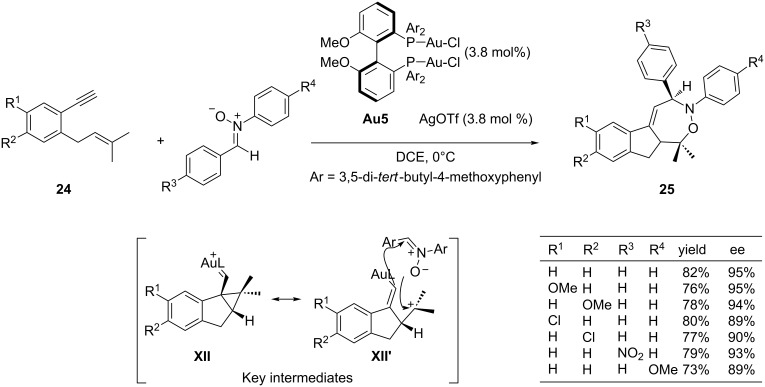
Enantioselective formal [4 + 3] cycloadditions leading to 1,2-oxazepane derivatives.

The gold-bound allyl cation species generated from allenes could be formally viewed as 1,2 or 1,3-zwitterionic cycloaddition components. In a recent paper, Liu and co-workers demonstrated that 1,6-enynes like **24** when treated with appropriated gold complexes lead to related 1,4-zwitterionic homologs that can be efficiently intercepted by nitrones in a formal [4 + 3] cycloaddition reaction. The resulting 1,2-oxazepane derivatives **25** are isolated as single diastereoisomers with high enantioselectivities (84–95% ee) [81]. Optimal conditions for the enantioselective variant of the process involved the use of the chiral gold complex **Au5** (3.8 mol %), derived from MeO-DTBM-Biphep, in combination with AgOTf. The requirement of equimolar amounts of the silver salt and the bisgold complex suggests that only one Au–Cl bond remains intact in the gold catalyst (Scheme 16). From a mechanistic point of view, the authors proposed that the intermediate species can be viewed as either a cyclopropylgold carbenoid **XII** or as a zwitterionic alkenylgold derivative **XII’**, the latter being the species that participates in the cycloaddition to the nitrone, probably in a concerted pathway, and thus providing the corresponding products with high levels of stereoselectivity [82–83].

**Scheme 17:**
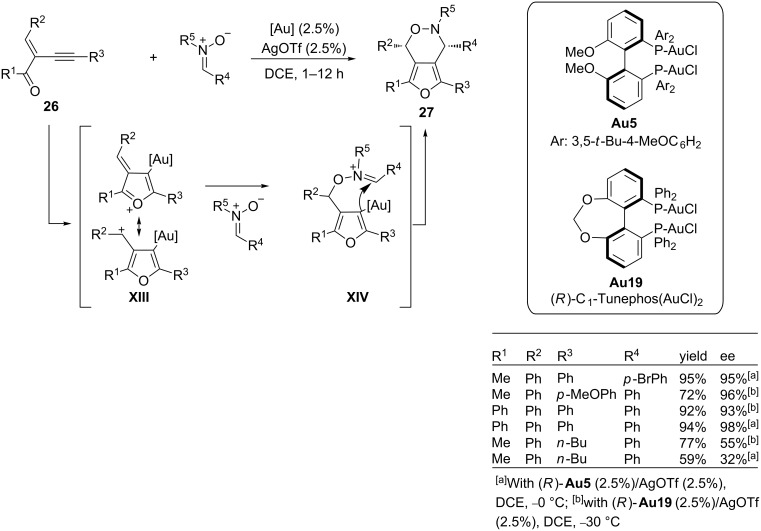
Enantioselective gold(I)-catalyzed 1,3-dipolar [3 + 3] cycloaddition between 2-(1-alkynyl)-2-alken-1-ones and nitrones.

Previously, in 2009, J. Zhang had reported a gold(I)-catalyzed 1,3-dipolar [3 + 3] cycloaddition between 2-(1-alkynyl)-2-alken-1-ones **26** and nitrones. The reactions provide fused heterobicyclic oxazine derivatives of type **27** with good yields and excellent regio- and diastereoselectivities. The racemic series, promoted by Ph_3_PAuCl/AgOTf as a catalyst [84], was translated into an enantioselective version by using any of the chiral bisgold complexes derived from (*R*)-C1-Tunephos (**Au19**) or (*R*)-MeO-DTBM-Biphep (**Au5**) (Scheme 17) [85]. From a mechanistic point of view, the authors proposed the generation of a zwitterionic furanylgold complex of type **XIII** by a gold-promoted intramolecular cyclization process. This key intermediate is then trapped by the nitrone to afford **XIV**, which eventually evolves to the product by an intramolecular cyclization (Scheme 17).

**Scheme 18:**
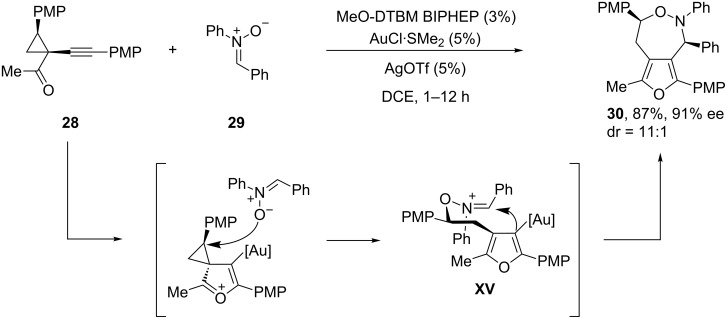
Enantioselective [4 + 3] cycloaddition leading to 5,7-fused bicyclic furo[3,4-*d*][1,2]oxazepines.

Finally, the same group recently reported a related reaction of 1-(1-alkynyl)cyclopropyl ketones such as **28**, by means of a gold(I)-catalyzed asymmetric [4 + 3] cycloaddition. In this article, the authors demonstrated that a chiral gold catalyst generated from MeO-DTBM-Biphep was able to promote the [4 + 3] cycloaddition between ketone **28** and the nitrone **29**, to afford the corresponding 5,7-fused bicyclic furo[3,4-*d*][1,2]oxazepine **30** in good yield, high diastereoselectivity (dr 11:1) and excellent 91% ee (Scheme 18) [86–88]. However, the extension of this method to 1-(1-alkynyl)cyclopropyl ketones and nitrones others than **28** and **29** remains to be demonstrated.

## Conclusion

In the last years there have been remarkable advances in the development of enantioselective gold(I)-catalyzed cycloadditions. Although the area is still in its infancy, there is enough evidence to predict that the number of examples will steadily increase in the near future. It would be highly desirable to have more information on the precise factors that govern the enantioselectivity process, as this will facilitate the design of new asymmetric processes.
